# Histopathological and Ultrastructural Observations of *Zanthoxylum armatum* Infected with Leaf Rust Causal Agent *Coleosporium zanthoxyli*

**DOI:** 10.3390/jof11110809

**Published:** 2025-11-14

**Authors:** Xikun Kang, Jingyan Wang, Wenkai Hui, Wei Gong

**Affiliations:** Forest Ecology and Conservation in the Upper Reaches of the Yangtze River Key Laboratory of Sichuan Province, College of Forestry, Sichuan Agricultural University, Chengdu 611130, China

**Keywords:** plant–pathogen interaction, ultrastructure, fluorescence microscopy, rust disease, host response

## Abstract

The fungus *Coleosporium zanthoxyli* is the causal agent of leaf rust in Chinese prickly ash pepper (*Zanthoxylum armatum* ‘Hanyuan putaoqing’), seriously impacting its industrial development. However, little is currently known about the infection and pathogenesis of *C. zanthoxyli* on *Z. armatum*. In this study, the infection of *Z. armatum* by *C. zanthoxyli* was reported at histological and cytological levels by a fluorescence microscope and transmission electron microscopy (TEM) for the first time. Fluorescence microscopy with fluorophore Alexa 488 (WGA-FITC) stained samples revealed that the infection process comprised three distinct stages: penetration (0–1 days post inoculation, dpi), parasitic growth (3–5 dpi), and sporulation (≥7 dpi). The number of haustoria increased during the osmotic and parasitic periods and then decreased; the length of hyphae also increased rapidly and then decreased. TEM analysis during these stages demonstrated that as disease severity increased, chloroplasts and mitochondria enlarged significantly, accompanied by a marked accumulation of starch granules and osmiophilic granules. At later stages, the nuclei became irregular, the grana lamellae were blurred, and the lamellar structure was arranged disorderly, and leaf tissues were extensively colonized by fungal hyphae and haustoria, leading to cellular necrosis and distorted cell walls. Notably, the sporulation phase was characterized by dense rust spore clusters covering the leaf surface. These findings provide critical insights into the ultrastructural changes induced by *C. zanthoxyli* during infection, elucidating key mechanisms of rust-induced damage in Chinese prickly ash and identifying the parasitic phase as a critical window for control strategies. This study lays a foundation for further research on rust pathogenesis and the development of Chinese prickly ash targeted control strategies.

## 1. Introduction

Plants face continuous threats from pathogens during growth, and disease emergence reflects complex interactions between pathogens and hosts, shaped by long-term co-evolution [1,2]. Investigating these plant–pathogen interactions is critical to elucidating pathogenic virulence mechanisms and host defense strategies from histology and cytology, laying the foundation for the scientific use of plant disease resistance [3]. Rust is one of the most devastating globally [4], affecting many important economically valuable crops and trees, such as wheat, soybean, poplar, and eucalyptus, causing significant economic and ecological losses [5,6,7,8]. Rust fungi are obligate biotrophs that establish parasitic associations with their host plants [6]. Previous studies have investigated the relationship between rust fungi and host plants at the histological and cytological levels. Silva et al. [9] used optical and transmission electron microscopy to examine coffee leaf rust (*Hemileia vastatrix* Berk and Br.) and associated cell death processes. Ayliffe et al. [10] observed the development process of rust fungi on rice leaves by fluorescence staining. Similarly, fluorescence electron microscopy observed the development process of stripe rust in wheat and subcellular changes in host–pathogen interactions during the seedling and boot stages were analyzed by transmission electron microscopy [3]. The process of *Melampsora larici-populina* on poplar leaves has been observed by using scanning and transmission electron microscopes [11]. Additionally, optical microscopy and scanning electron microscopy evaluated the pre-penetration processes of *Puccinia psidii* in leaves at different phenological stages of a susceptible clone (3918) of *Eucalyptus grandis* [12]. Fluorescence and confocal microscopy are useful for studying fungal growth patterns and host responses at the cellular level [3,10,13,14,15,16]. Observing the growth and development and host cell changes in the process of pathogen infection provides a scientific basis for revealing the mechanism of plant disease resistance and rational use of host disease resistance to control the occurrence and development of diseases, and it also lays a theoretical foundation for investigating the molecular mechanism underlying the pathogenesis of disease.

Chinese prickly ash belongs to the *Zanthoxylum* genus, including *Zanthoxylum armatum* DC. and *Zanthoxylum bungeanum* Maxim. [17], and belongs to the Rutaceae family; it is an economically valuable spice, medicinal plant, and woody oil tree species [18,19,20]. *Z. armatum*, primarily cultivated in southwest China [21], exhibits strong drought resistance and adaptability, contributing to high economic and ecological benefits [22,23]. Currently, the cultivation area in China has reached 14 million hectares, with an economic output value of USD 2 billion [24]. However, rust is a catastrophic disease in the production of Chinese prickly ash [25], and rust disease is caused by the *Coleosporium Zanthoxyli* Diet. et Syd. [25,26]; it severely threatens *Z. armatum* production, with infection rates reaching 100% and defoliation exceeding 90%, ultimately reducing yield by over 20% and hindering industry development [25,27].

While existing research on *Z. armatum* rust has revealed transcriptomic and metabolomic differences between resistant and susceptible varieties [25] and identified the role of salicylic acid in spore inhibition and induced resistance [26], little is known about the cellular and ultrastructural dynamics of the infection process. The main aim of this study was to document the histological characteristics of infection by *C. zanthoxyli* on *Z. armatum* leaves, thereby gaining a clearer understanding of the pathogen’s invasion strategy. These results not only provide a theoretical basis for further molecular-level research on the pathogenic mechanism of *C. zanthoxyli* but also offer a scientific foundation for its prevention and control.

## 2. Materials and Methods

### 2.1. Experimental Site

The experimental plots were established at the Chongzhou teaching and research experimental site located in Sichuan Province, China (103°38′ E, 30°35′ N). The environment and conditions are described by Hui et al. [28]. Soil chemical properties were as follows: pH, 7.06; organic matter, 18.72 g·kg^−1^; total N, 1.46 g·kg^−1^; total P, 0.31 g·kg^−1^; total K, 21.4 g·kg^−1^; alkali-hydrolyzed N, 130.0 mg·kg^−1^; available P, 14.3 mg·kg^−1^; and available K, 92.8 mg·kg^−1^.

### 2.2. Plant Material

*Zanthoxylum armatum* ‘Hanyuan putaoqing’ (authenticated by the Sichuan Province Forestry Variety Certification Committee, SV-ZA-002-2018 [29]) was used in this study. Plants were spaced at 2 m × 3 m and established in spring 2016.

### 2.3. Inoculation of Z. armatum Leaves with C. zanthoxyli

The pathogenic fungus *C. zanthoxyli* was collected from naturally occurring Chinese Pepper rust leaves in a Chinese Pepper plantation located in Chongzhou City, Sichuan Province. Spores were stored at 4 °C, and the spore suspension was adjusted to 1 × 10^8^ spores mL^−1^ with sterilized water. Healthy, uninoculated plants of the same cultivar, age, and similar size were maintained alongside the inoculated plants to serve as controls, and they were cultivated under identical environmental conditions and received uniform field management practices, with the sole exception of the inoculation treatment. Branches of the trees were selected from four directions (east, west, north, and south) in the inoculated area, and the spore suspension was evenly sprayed over their front and back surfaces using a spray bottle and moisturized for 24 h. The uninoculated area was sprayed with water only as a control and treated in the same way. Isolation rows were used to separate the inoculated and uninoculated areas, with the application of a rust-controlling drug on the periphery serving as a barrier. Each treatment was replicated three times, with five plants per replication, for a total of 30 plants.

### 2.4. Leaf Sampling

Leaf samples were randomly chosen from each tree, selecting the 4th to 6th leaves from the top downwards on branches facing four directions (east, west, north, and south). Leaves were collected on the day before inoculation (−1 dpi) and 1, 3, 5, 7, 14, 21, 30, 45 and 60 days post inoculation (dpi). The collected leaves from each replicate were combined for testing, and the sample from the uninoculated area underwent the same procedure. Subsequently, the leaves were photographed, placed in a fresh-keeping box, and transported back to the laboratory for future analysis. Each experiment was repeated three times.

### 2.5. Histopathology of the Interaction Between Z. armatum and C. zanthoxyli

#### 2.5.1. Physical Observation of Leaf Rust Symptoms and Signs, and Molecular Biology and Phylogenetic Identification

To preserve the collected leaves and to observe the signs of rust on the leaves, photographs were taken of both the upper and lower surfaces of the leaves. The morphology, size, and color of the spore masses on the leaves were examined under an Olympus SZX16 microscope (Olympus, Tokyo, Japan). Isolated single spores were obtained from the leaf samples and examined under 40× magnification of an Olympus BX-51 microscope (Olympus, Tokyo, Japan).

Genomic DNA was extracted from the collected urediniospores of the rust pathogen using a DNA extraction kit (Beijing Qingke Biotechnology Co., Ltd., Beijing, China). The internal transcribed spacer (ITS) region was amplified with the universal primers ITS1 (5′-TCCGTAGGTGAACCTGCGG-3′) and ITS4 (5′-TCCTCCGCTTATTGATATGC-3′). The PCR reaction procedures included pre-denaturation at 98 °C for 2 min, denaturation at 98 °C for 10 s, annealing at 53 °C for 30 s, extension at 72 °C for 10 s, for a total of 35 cycles, and final extension at 72 °C for 5 min. The amplified products were detected by agarose gel electrophoresis; the PCR products were sequenced by the Qingke Biotechnology Company (Beijing, China) and assembled/trimmed. The resulting sequences were aligned via BLAST on NCBI (https://www.ncbi.nlm.nih.gov/, accessed on 11 November 2025) and a phylogenetic tree was constructed in MEGA 12 using the Neighbor-Joining method (1000 bootstrap replicates, p-distance model).

#### 2.5.2. WGA-FITC Fluorescence Staining

According to the method of Ayliffe et al. [10], the leaves were selected at 1, 3, 5, 7, 14 and 30 dpi of *C. zanthoxyli* inoculation. They were cut into 2 cm pieces and placed in a centrifuge tube containing 10 mL of 1 mol·L^−1^ KOH (Xilong Scientific, Shantou, Guangdong, China) + 15 μL Silwet L-77 solution (Sigma, St. Louis, MO, USA) for 24 h. Subsequently, the leaves were washed three times with 50 mmol·L^−1^ Tris (pH 7.5) buffer (Solarbio, Beijing, China), each time with a 10 min interval. The leaves were then transferred to a glass Petri dish. Afterward, the leaves were stained with WGA at 20 µg mL^−1^ conjugated to fluorophore Alexa 488 (WGA-FITC, Sigma, St. Louis, MO, USA) and stored in a refrigerator at 4 °C for 24 h. Then, they were rinsed twice with distilled water (15 min/wash), and the washed leaves were mounted on a glass slide for microscopy with the Olympus BX-51 fluorescence microscope (Olympus, Tokyo, Japan).

#### 2.5.3. Transmission Electron Microscopy

Leaves from *Z. armatum* inoculated with rust fungi were selected at −1, 1, 3, 5, 7, 14 and 30 dpi, cut into 1 mm × 1 mm, placed in 2.5% Gluta fixative, and stored at 4 °C for 24 h. The samples were then sent to the Lilai Biomedicine Experiment Center (Chengdu, China). The samples, during processing, were post-osmicated, dehydrated through a graded ethanol series, and embedded in epoxy resin. After polymerization at 60 °C for 24 h, ultrathin sections (50 nm) were prepared, stained with uranyl acetate and lead citrate, and examined using JEM-1400PLUS TEM (JEOL Ltd., Tokyo, Japan).

### 2.6. Statistical Analyses

All experiments included three independent biological replicates. For histopathological and histochemical observations, a minimum of 50 infection sites from eight to ten leaf sections were analyzed per time point. Prior to analysis, all quantitative data (including hyphal length, haustorium counts, organelle counts, and organelle damage rates) were assessed for normality using the Shapiro–Wilk test and for the homogeneity of variances using Levene’s test. Data meeting the assumptions of normality and homoscedasticity were subsequently analyzed by one-way analysis of variance (ANOVA) in SPSS 26.0, with the significance level set at α = 0.05. Where ANOVA indicated significant differences, Duncan’s multiple range test was applied for post-hoc comparisons among groups. The figures were processed with Origin 2025.

## 3. Results

### 3.1. Symptoms of Leaf Rust on Z. armatum

**Figure 1:**
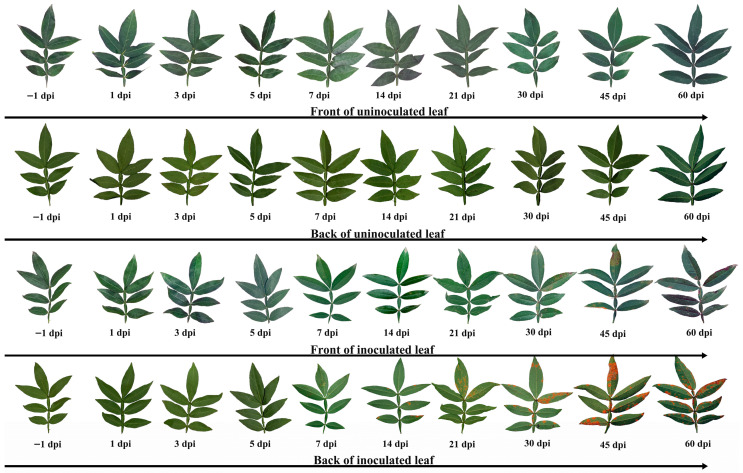
Rust phenotypes of *Z. armatum* leaves at different stages post inoculation of *C. zanthoxyli*.

The progression of leaf rust symptoms on *Z. armatum* showed distinct temporal patterns (Figure 1). At 1–5 dpi, no visible disease symptoms appeared on the leaves inoculated with the pathogens. Initial symptoms appeared at 7 dpi, characterized by chlorotic spots on the adaxial leaf surface and scattered urediniospore pustules (yellow powder masses) on the abaxial surface. At 14 dpi, brown dead classes appeared on the adaxial surface of the leaf blade, and more and more urediniospore piles appeared on the back of the leaves, forming a ring-shaped urediniospore pile while the surrounding leaves were discolored; at 21 dpi, this was accompanied by circular necrotic spots on the upper surface and clustered urediniospore pustules on the lower surface. At 30 dpi, there was a large area of dark brown necrotic spots on the front of the leaves surrounded by yellow, and a large number of urediniospore piles appeared on the back of the leaves and petioles, most of which were circular integrated circles and on the petiole, and at the base of the urediniospore pile, teliospore piles were producing brown substances, and the diseased leaves began to fall off. At 45 dpi, there was a large area of necrotic spots on the front of the leaves, the yellow color deepened around them, and the shape of the leaves was deformed, and there were patches of urediniospores and teliospores on the back of the leaves, even on the petiole. At 60 dpi, the leaves showed black necrotic spots on the front of the leaves, the leaves were deformed and curled, and a large number of leaves fell off. The urediniospore pile was completely transformed into a brown colloid teliospore pile and covered the back of the leaves.

### 3.2. Morphology and Development of Rust Fungi

**Figure 2:**
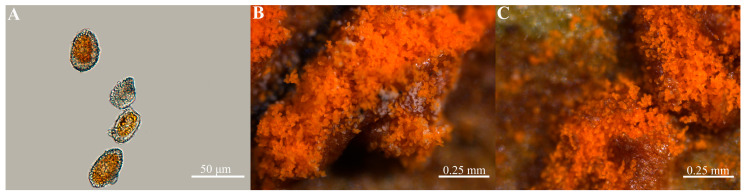
The microscope of imagination of spores of *Coleosporium zanthoxyli*. (**A**) Morphological characteristics of rust pathogen under ×40 microscope; (**B**) Morphological characteristics of urediniospores under stereomicroscopy; (**C**) Morphological characteristics of teliospores under stereomicroscopy.

Under a ×40 optical microscope, examination revealed distinct morphological characteristics of the urediniospores (Figure 2). The urediniospores of the rust fungus on the leaves of *Z. armatum* were yellow or yellow-brown and generally had an oval or round shape, with a colorless wall, which surrounded the yellow spore portion covering its surface uniformly, and the warts were colorless and transparent (Figure 2A). The size of 50 urediniospores was measured randomly; the maximum transverse diameter was 48.75 μm, the minimum was 22.57 μm, and the average was 35.07 μm, and the maximum longitudinal diameter was 27.62 μm, the minimum was 17.80 μm, and the average was 22.71 μm. Under a stereomicroscope, the urediniospores were scattered on the back of the leaves and were yellowish hyaline and long ellipsoidal when fresh and white when faded (Figure 2B); the teliospores in the teliospore pile were columnar, and the single teliospores on the fresh leaves were brown with transparent colloids, which were reddish brown when aggregated (Figure 2C).

The ITS1 and ITS4 primers were used to amplify the pathogen of Chinese prickly ash pepper rust by PCR, and the PCR products were observed by agarose electrophoresis. PCR gel electrophoresis pictures are presented in Supplementary Figure S1, and a DNA fragment of about 500 bp was amplified, and an rDNA-ITS sequence of 518 bp was obtained by sequencing and assembled. The data reported in this paper have been deposited in the GenBase [30] in National Genomics Data Center [31], Beijing Institute of Genomics, Chinese Academy of Sciences/China National Center for Bioinformation, under accession number C_AA126367.1 that is publicly accessible at https://ngdc.cncb.ac.cn/genbase. BLAST analysis indicated that it had the highest homology with *Coleosporium zanthoxyli* (GenBank Accession No. JQ219672.1) with 100% query coverage, 99.81% identity, and an E-value of 0. Phylogenetic analysis further confirmed that the obtained sequence clustered robustly within a clade containing *C. zanthoxyli* reference strains (Figure S2). Together with the morphological characteristics observed previously, these molecular results unequivocally identify the causal agent of the *Z. armatum* rust as *Coleosporium zanthoxyli* Diet. et Syd.

**Figure 3:**
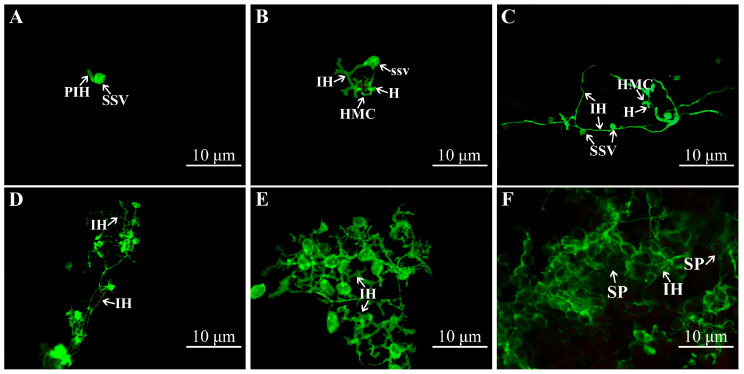
Infection features of *C. zanthoxyli* at a series of time points post-inoculation. Leaves were examined under a fluorescence microscope after staining with fluorophore Alexa 488 (WGA-FITC). (**A**) 1 dpi; (**B**) 3 dpi; (**C**) 5 dpi; (**D**) 7 dpi; (**E**) 14 dpi; (**F**), 21 dpi; SSV, substomatal vesicle; PIH, primary infected hyphae; IH, infected hyphae; HMC, haustorial mother cell; H, haustorium; SP, urediniospore.

The infection process of *C. zanthoxyli* was studied microscopically after staining fungal structures with WGA-FITC, as shown in Figure 3. Urediniospores entered the stomatal pore, and then the substomatal vesicle was formed inside the stomatal cavity, which further differentiated into primary hyphae at 1 dpi in infected leaves (Figure 3A). Haustorium formation was observed at 3 dpi (Figure 3B). At 5 dpi, the infected hyphae spread between mesophyll cells and the haustorium increased, with rust fungi obtaining nutrients from mesophyll cells, and their intercellullar hyphae grew fast, ultimately forming a colony structure (Figure 3C). At 7 dpi, a significant increase in the number of fungal hypha branches and enhanced hyphal growth was observed; the colony structures were intertwined, and the sporogenous cells continued to differentiate to form urediniospores (Figure 3D). At 14 dpi, a substantial aggregation of sporogenous hyphae was observed, and the sporogenous cells continued to differentiate, giving rise to urediniospores that accumulated to form a spore stack; concurrently, the mycelium’s growth appeared to be retarded (Figure 3E). At 30 dpi, a substantial number of spore piles had formed, while the hyphae exhibited poor development and failed to grow further (Figure 3F).

**Figure 4:**
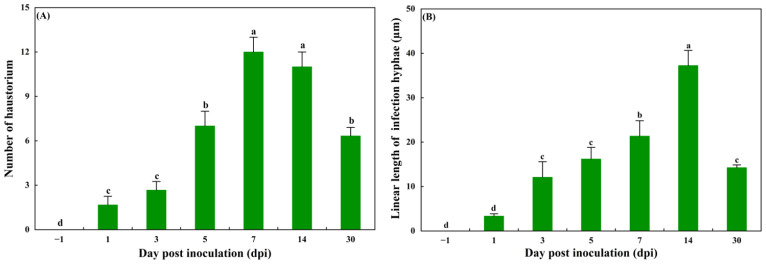
The linear length of infected hyphae (syn. colony length) (from the substomatal vesicle to the apex of the longest infected hypha), and the number of haustoria. (**A**) the linear length of infected hyphae; (**B**) the number of haustoria. Mean values from three independent replications and vertical bars represent the standard deviations. Different small letters indicate statistically significant differences (Duncan’s test, *p* < 0.05).

The linear length of infected hyphae and the number of haustoria per infection unit at a series of time points post inoculation were also measured, and they first increased and then decreased (Figure 4).

### 3.3. Ultrastructural Changes in Leaf Cells

**Figure 5:**
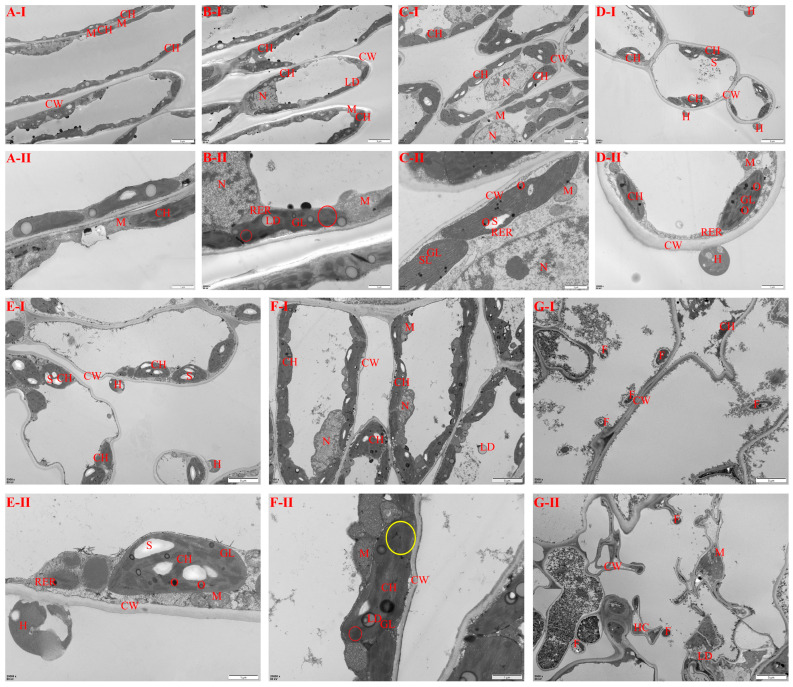
Ultrastructure of leaves of *Z. armatum* inoculated with rust fungus (**A**–**G**). Ultrastructure of *Z. armatum* leaves at −1, 1, 3, 5, 7, 14 and 30 dpi. N, nucleus; CH, chloroplast; M, mitochondrion; CW, cell wall; LD, lipid droplet; RER, rough endoplasmic reticulum; GL, grana lamellae; SL, stromal lamellae; O, osmiophilic granules; S, starch granules; H, Haustorium; HC, intercellular hyphae; F, Fungus spores; ○, blurred basal lamellae; ○, disordered structural alignment of lamellae. Bars: (**I**,**G-II**): 5 μm; (**A-II**–**F-II**): 1 μm.

The structure of the cells in *Z. armatum* leaves, which had been inoculated with rust fungus, was observed in further detail using a transmission electron microscope. This observation primarily included the morphological changes in parenchyma cells in leaf palisade tissue and the adhesion of fungi to the cell wall (Figure 5).

The cell structure of healthy leaves is normal, and the cytoplasm and organelles in the cells are evenly arranged and distributed. There is also a large number of chloroplasts regularly arranged on the plasma membrane. The chloroplast bilayer membrane is complete, the lamellar structure is clear, and the arrangement is orderly. The mitochondria are abundant, the outer membrane structure is complete, and the cristae formed by the invagination of the inner membrane are clear (Figure 5(A-I,A-II)).

The ultrastructure of *Z. armatum* leaves at 1 dpi is shown in Figure 5(B-I,B-II). The chloroplasts were fusiform-shaped and were arranged along the plasma membrane. A few lipid droplets were embedded in the chloroplasts, and the boundaries of the chloroplast membrane appeared blurred. The thylakoids in the chloroplast displayed blurring and a disorganized arrangement of the stromal lamellae, and the granal lamellae were thinner. The structure of the mitochondria appeared slightly swollen and oval-shaped, with a clear crista structure and a reduction in the number of cristae. The matrix was homogeneous and had reduced electron density, while the outer membrane remained continuous. The rough endoplasmic reticulum appeared as parallel membranes, with narrow spaces between them and ribosomes attached to the surface.

At 3 dpi, numerous chloroplasts were present in the cytoplasm, with some chloroplasts appearing enlarged. Starch granules were observed within the chloroplasts, while a few osmiophilic granules were present in the stroma. The mitochondria were swollen and took on an oval shape, and the crista structures were clear but reduced in number (Figure 5(C-I,C-II)).

At 5 dpi, the microstructure of leaf cells changed significantly: the cytoplasm was sparse, with a few chloroplasts that were enlarged and contained a few starch grains; osmiophilic granules in the stroma were enlarged; mitochondria were enlarged and rounded, displaying a clear ridge structure, a uniform matrix, and a continuous outer membrane; the rough endoplasmic reticulum was parallel and membranous, featuring a narrow membrane space; and the haustorium was attached to the outside of the cell wall (Figure 5(D-I,D-II)).

At 7 dpi, the chloroplasts and starch granules became larger; there were more osmiophilic small granules with lower electron density in the matrix; mitochondria were oval, with a clearer crista structure and a more homogeneous matrix; more haustoria were attached outside the cell wall; and some haustoria ruptured (Figure 5(E-I,E-II)).

At 14 dpi, the nuclei were irregular and the chromatin was distributed evenly, mainly in the euchromatin, and a small amount of lipid droplets were embedded in the chloroplasts; the chloroplasts were swollen into a rounded shape, and the starch granules and osmiophilic granules were enlarged; there were many grana lamellae in the thylakoid and the grana stack was thin, the grana lamellae were blurred, and the lamellar structure was arranged disorderly; and the mitochondrion was an elongated ellipsoid, with a blurred crista structure, the basal lamellae were evenly distributed and were a uniform greyish-black color, and the adventitia was continuous (Figure 5(F-I,F-II)).

At 30 dpi, leaf cells were necrotic, with a lost cytoplasm, the number of chloroplasts was small and fusiform, the electron density was high, and the outer membrane structure was unclear; some of the thin-walled cells were crumpled, the morphology of the cell wall was distorted, and the cells were filled with spores and hyphae (Figure 5(G-I,G-II)).

**Figure 6:**
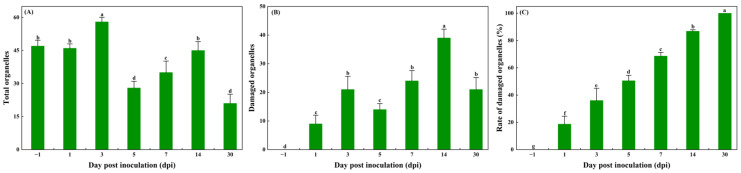
Analysis of damaged organelles in leaves by transmission electron microscopy. (**A**) total organelles; (**B**) damaged organelles; (**C**) rate of damaged organelles.Based on TEM observation, organelles were classified as damaged when showing evident ultrastructural alterations, including swelling, disintegrated membranes, or lytic damage. The percentages of damaged organelles = the number of damaged organelles/the total number of organelles × 100%. Data are presented as mean ± SD; different small letters indicate statistically significant differences (Duncan’s test, *p* < 0.05).

For the statistical analysis, the percentage of damaged critical organelles in the leaves of *Z. armatum* was significantly different at different inoculation stages (Figure 6).

## 4. Discussion

As an important fungal disease, leaf rust caused by *C. zanthoxyli* shows severe disease symptoms with yellow urediniospores on leaf surfaces, which greatly influences the production and economic value of *Z. armatum*, but the process of fungal *C. zanthoxyli* infection is not known. This study provides the first comprehensive microscopic analysis of *C. zanthoxyli* infection in *Z. armatum*, combining WGA-FITC fluorescence microscopy and transmission electron microscopy to elucidate the histopathological changes during disease progression. Consistent with previous descriptions [32,33,34,35], symptoms in our study included hypophyllous urediniospores that appeared as circular, gregarious, or scattered orange-yellow erumpent pustules, with disease severity progressively increasing over time. This symptom progression aligns with findings from studies on Chinese prickly ash pepper, where *C. zanthoxyli* infection also led to characteristic yellow urediniospores and leaf defoliation. Notably, late infection stages were characterized by dark brown necrotic spots on the adaxial leaf surface and dense spore accumulation on the abaxial side, ultimately resulting in premature defoliation.

While *C. zanthoxyli* exhibits a narrow host range specific to Chinese prickly ash pepper, its infection process shares remarkable similarities with other economically important rust fungi. Similarly to the wheat stripe rust fungus (*Puccinia striiformis*) [36] and poplar rust (*Melampsora larici-populina*) [37], the infection cycle of *C. zanthoxyli* can be divided into three distinct developmental stages: penetration (0–1 dpi), parasitic growth (3–5 dpi), and sporulation (≥7 dpi) [36,37,38]. Histological and cytological observations showed that during the penetration stage, the fungus forms substomatal vesicles in the substomatal chambers, a critical step also observed in wheat stripe rust infection [36]. The formation of substomatal vesicles marks the successful infection of rust fungi, and the transition from a spore to a hypha marks the onset of pathogenicity, enabling the fungus to invade host leaves via open stomata [39,40]. During the parasitic growth phase, we observed extensive hyphal colonization and haustorium formation, representing the establishment of the host–pathogen interface. The haustorium, as the primary site for nutrient uptake and molecular dialog between the pathogen and host [41,42,43], shows structural and functional conservation across different rust species. Quantitative analysis revealed that both hyphal extension and haustorial production peaked during this critical period (3–5 dpi) before declining as the fungus transitioned to sporulation (Figure 4).

The appearance of necrotic spots indicated that *C. zanthoxyli* successfully invaded and colonized host tissues, although host cells were differentially affected within the inoculation site depending on the distance from the invading hyphae, and the gradual degradation of organelles proved this [44]. Similar individual changes in the ultrastructure of host cells of infected plants were observed in other pathological systems during viral, bacterial, and fungal infection, such as cell lysis, the decomposition of nuclei and chloroplasts, and the presence of osmophilic granules [45,46,47,48]. Understanding the fungal infection process can guide strategic decisions regarding the timing of fungicide applications and other control measures. The early penetration and parasitic phases (0–5 dpi) represent a critical window for control measures, as the fungus is establishing biotrophy and remains vulnerable to systemic fungicides that inhibit metabolic or morphogenetic processes. The marked increase in hypha growth and haustorium formation during this period (Figure 4) underscores the importance of intervening before the fungus reaches its peak biomass and sporulation capacity. Once sporulation has initiated (≥7 dpi), the integrity of leaf tissues is substantially compromised, and control efficacy may decline. Therefore, monitoring early symptom development and applying targeted antifungals prior to 5 dpi could effectively suppress rust establishment and minimize yield loss.

Leaf cells play an important role in photosynthesis and are also a key component of resistance to fungal pathogen invasion and expansion [49]. Our ultrastructural observations revealed significant organellar alterations, particularly in chloroplasts, which underwent swelling, the disorganization of grana lamellae, and the accumulation of starch and osmiophilic granules. Research on the wheat stripe rust fungus (*Puccinia striiformis*) has revealed that certain effectors, such as Pst_TTP1, are secreted into the host cell to actively suppress chloroplast-mediated defense responses [50]. For instance, Pst_TTP1 targets the chloroplast thioredoxin TaTrx, inhibiting a pro-senescence signaling cascade (the TaTrx–TaSGR1 module) that would normally generate ROS and trigger protective cell death around the infection site [50]. By suppressing this pathway, the rust fungus maintains host cell viability, thereby ensuring a continuous nutrient supply—a strategy characteristic of biotrophic pathogens [51]. The “green island” phenomenon often associated with rust infections is a direct visual consequence of this manipulation, wherein chlorophyll is retained in infected tissues while surrounding areas undergo senescence [50]. Similar chloroplast-targeting mechanisms have been well characterized in wheat stripe rust, and our observations of chloroplast swelling and disorganized grana are consistent with such effector-mediated manipulation. But their existence in *C. zanthoxyli* remains to be experimentally verified.

At advanced infection stages (14 dpi), we observed cell nuclear irregularity and chromatin condensation. The cell nucleus is another organelle that responds to fungal infection [52]. The interaction between fungal pathogens and susceptible plants can destroy the nuclear structure of plants and change the form of chromatin condensation [53,54]. This phenomenon may be linked to pathogenic strategies aimed at reprogramming host gene expression. Some studies have shown that certain fungal pathogens secrete effector proteins that interfere with nuclear processes to suppress plant immunity [55]. The nuclear alterations observed in this study may reflect such interference, potentially compromising the expression of defense-related genes. Chloroplasts play a crucial role in plant immunity to pathogens, and increasing evidence suggests that plant pathogens use chloroplast homeostasis as a pathogenic mechanism [56,57]. Chloroplasts are a kind of organelle susceptible to environmental changes [58]. When plants are stressed, the chloroplasts are affected to some extent, and their structural changes affect the degree of thylakoid accumulation, and the number of starch granules and osmiophilic granules change adaptively with the environment [59,60]. In this study, starch granules and osmiophilic granules were significantly accumulated in chloroplasts, indicating that chloroplasts responded to rust infection. When chloroplast structure was destroyed, photosynthesis decreased, and photosynthetic products were deposited in chloroplasts in the form of starch [61,62]. The substantial accumulation of starch granules likely reflects a severe impairment of photoassimilate export, which may be a direct result of the fungal invasion creating a metabolic “sink” that diverts carbon resources toward the infection site to support fungal growth [50]. Osmophilic granules are the dynamic lipid pool of the thylakoid membrane, and if the lipid in the thylakoid membrane is too high, osmiophilic granules will absorb the lipid and increase. The osmiophilic granules are the result of chloroplast thylakoid membrane degradation, and the number and size of osmiophilic granules can also be used as an indicator of thylakoid disintegration [63]. Under pathogenic stress, the production of reactive oxygen species (ROS) can induce lipid peroxidation, and osmiophilic granules may function as reservoirs for damaged membrane lipids, reflecting the breakdown of the photosynthetic apparatus [64]. However, the underlying process behind chloroplast degradation during infection is complex and requires further extensive research.

In summary, the findings of this study depict a coordinated pathogenic attack. Future research should prioritize the identification of specific fungal effectors in the *Z. armatum*–rust pathosystem and their host targets, particularly those localized within chloroplasts. A deeper understanding of these mechanisms—especially how the pathogen modulates ROS signaling and host cell death—will be crucial for developing novel control strategies. Such strategies may include the use of gene editing to disrupt effector targets or to enhance the chloroplast’s role as a defense signaling organelle. This will help elucidate the infection mechanism of the rust fungus and its interaction with the host, thereby contributing to the development of more effective disease control strategies.

## 5. Conclusions

This study provides the first comprehensive cytological characterization of the infection process of *Coleosporium zanthoxyli* on *Zanthoxylum armatum*, establishing a three-stage model of pathogenesis comprising penetration (0–1 dpi), parasitic growth (3–5 dpi), and sporulation (≥7 dpi). Through the integrated application of fluorescence and transmission electron microscopy, we demonstrate that each developmental stage is associated with specific and progressive ultrastructural pathologies in host cells, including organellar enlargement, the aberrant accumulation of starch and osmiophilic granules, and the progressive disintegration of cellular integrity. Our findings identify the parasitic growth phase (3–5 dpi) as a critical stage for disease establishment, where extensive hyphal colonization and haustorium formation facilitate nutrient diversion and cause irreversible cellular damage. These observations provide a mechanistic basis for the necrosis of chlorosis and photosynthetic decline in infected plants. In addition, we identified the early to mid-infection phase (0–5 dpi) as a key window for intervention and suggested that control measures, especially the use of fungicides, should be taken in a timely manner before widespread colonization and sporulation occur for strategic measures to disrupt the establishment of fungi. These insights advance our understanding of *C. zanthoxyli* pathogenicity and provide a cytological foundation for future research. Subsequent investigations should focus on (1) identifying and characterizing specific fungal effectors responsible for manipulating host cell processes; (2) elucidating the molecular mechanisms underlying organellar dysfunction and host defense responses; and (3) exploring genetic engineering or breeding strategies targeting the critical host–pathogen interfaces identified in this study. These investigations will be essential for developing durable resistance strategies against this economically significant pathogen.

## Data Availability

The original contributions presented in this study are included in the article/Supplementary Material. Further inquiries can be directed to the corresponding authors.

## Supplementary Materials

The following supporting information can be downloaded at https://www.mdpi.com/article/10.3390/jof11110809/s1, Figure S1: PCR–gel electrophoresis of the pathogen of *Z. armatum* rust; Figure S2: Phylogenetic tree.
