# Supplementary figures and images for: Histopathological and Ultrastructural Observations of Zanthoxylum armatum Infected with Leaf Rust Causal Agent Coleosporium zanthoxyli
Source: J Fungi (Basel). 2025 Nov 14;11(11):809. doi: 10.3390/jof11110809 (PMC12653885; doi:10.3390/jof11110809)

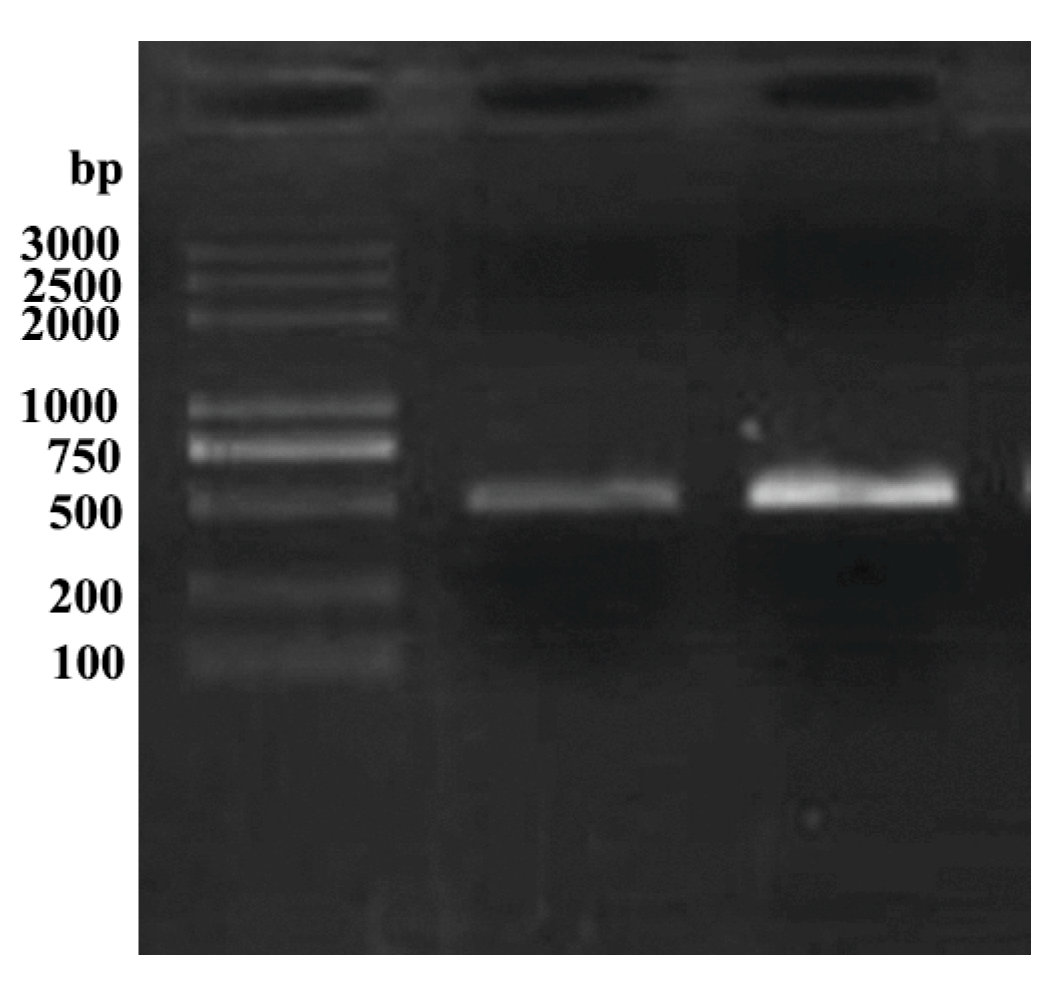

Supplement: Supplementary file 1 [file jof-11-00809-s001.zip › Figure S1.tif]

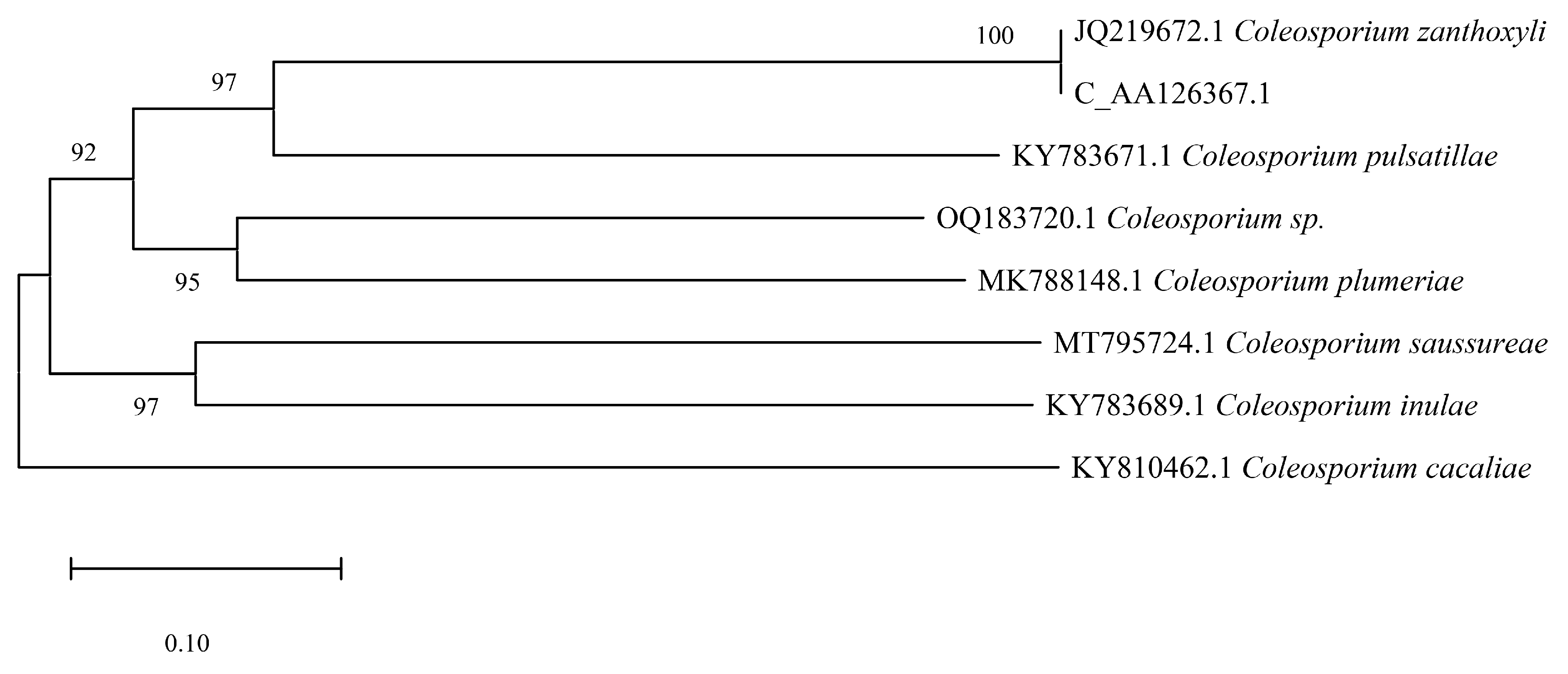

Supplement: Supplementary file 1 [file jof-11-00809-s001.zip › Figure S2.tif]
